# Efficacy and safety of microwave ablation and radiofrequency ablation in the treatment of hepatocellular carcinoma: A systematic review and meta-analysis

**DOI:** 10.1097/MD.0000000000029321

**Published:** 2022-07-29

**Authors:** Zhimin Dou, Fei Lu, Longfei Ren, Xiaojing Song, Bin Li, Xun Li

**Affiliations:** a The First Clinical Medical College of Lanzhou University, Lanzhou, China; b The Department of Critical Care Medicine of The First Hospital of Lanzhou University, Lanzhou, China; c The Second Department of General Surgery of The First Hospital of Lanzhou University, Lanzhou, China.

**Keywords:** hepatocellular carcinoma, microwave ablation, meta-analysis, radiofrequency ablation

## Abstract

**Background:**

Hepatocellular carcinoma (HCC) is one of the most common malignant tumors. Surgical resection is often only possible in the early stages of HCC and among those with limited cirrhosis. Radiofrequency ablation and Microwave ablation are 2 main types of percutaneous thermal ablation for the treatment of HCC. The efficacy and safety between these 2 therapy methods are still under a debate.

**Objective:**

To compare the efficacy and safety of Radiofrequency ablation and Microwave ablation in treating HCC.

**Methods:**

PubMed, EMBASE, the Cochrane databases and Web of Science were systematically searched. We included randomized controlled trials and cohort studies comparing the efficacy and safety of Radiofrequency ablation and Microwave ablation in HCC patients. Outcome measures on local tumor progression, complete ablation, disease-free survival, overall survival, or major complications were compared between the 2 groups. The random effect model was used when there was significant heterogeneity between studies, otherwise the fixed effect model was used.

**Results:**

A total of 33 studies, involving a total of 4589 patients were identified, which included studies comprised 7 RCTs, 24 retrospective observational trials, and 2 prospective observational trial. Microwave ablation had a lower local tumor progression than Radiofrequency ablation in cohort studies (OR = 0.78, 95% CI 0.64–0.96, *P* = .02). Complete ablation rate of Microwave ablation was higher than that of Radiofrequency ablation in cohort studies (OR = 1.54, 95% CI 1.05–2.25, *P* = .03). There was no significant difference in overall survival and disease-free survival between the 2 groups. Meta-analysis showed that there was no significant difference in the main complications between Microwave ablation and Radiofrequency ablation.

**Conclusions:**

Microwave ablation has higher complete ablation and lower local tumor progression than Radiofrequency ablation in the ablation treatment of HCC nodules. There was no significant difference in overall survival between the 2 therapy methods.

## 1. Introduction

Liver cancer is estimated to be ranked sixth on most currently diagnosed cancer as well as the fourth main reason of cancer death with about 841,000 new cases and 782,000 deaths occurred in 2018 worldwide.^[1]^ Hepatocellular carcinoma (HCC) accounts for the majority of primary liver cancers, and surgical resection is considered the gold standard of treatment for curative intent but is often only possible in the early stages of HCC and among those with limited cirrhosis.^[2]^ The multitude of available complimentary and additive locoregional therapies, which include trans arterial chemoembolization, percutaneous ethanol injection, Radiofrequency ablation (RFA), Microwave ablation (MWA), cryoablation, laser ablation, high-intensity focused ultrasound, and irreversible electroporation, encourage clinicians to implement a multidisciplinary treatment approach to improve the outcome of these patients.^[3]^ RFA and MWA are 2 main types of percutaneous thermal ablation.^[4]^ Despite several meta-analyses had compared MWA with RFA for the treatment of HCC,^[5–7]^ the efficacy and safety between these 2 modalities are still under debate, and also some new published studies were not included. Therefore, we performed a systematic review and meta-analysis of all the available randomized and observational studies to compare the efficacy and safety of RFA and MWA in treating primary HCC.

## 2. Methods

### 2.1. Search strategy

This systematic review and meta-analysis was conducted according to the Preferred Reporting Items for Systematic reviews and Meta-Analysis (PRISMA) guidelines.^[8]^ We searched for medical literature in electronic databases including The Cochrane Library, PubMed, Embase and Web of Science without any language and the publication date was before 31 December 2020. The search strategy included the following mesh terms or free text: “Radiofrequency ablation,” “radiofrequency therapy,” “microwave therapy,” “microwave ablation,” “hepatocellular carcinoma,” “liver cancer,” “hepatic cancer.” In addition, the references included were searched manually to avoid omitting any studies that met the inclusion criteria. Ethical approval and patient consent were not required, as this study was done on published data.

### 2.2. Selection criteria

Studies meeting the following criteria were selected:

1. Randomized controlled trials (RCTs), prospective or retrospective cohort studies compared efficacy and safety of MWA and RFA in HCC patients.
2. Outcome measures on local tumor progression (LTP), complete ablation (CA), disease-free survival, overall survival, or major complications compared between MWA and RFA for HCC were provided.
3. The studies were limited in humans.
4. The most complete and recent report of the trial was used when the same investigator reported data obtained from the same patients.

Duplicate publications, reviews, case reports, animal or cell experiments, and trials with incomplete data were excluded.

In our study, LTP was defined as any new lesion inside or adjacent to the ablated zone. CA was defined as no enhancement in ablated areas after ablation. Disease-free survival was defined as the length of time that patients survived without any signs of HCC after ablation. OS was defined as the length of time from the start of ablation to the date of the death or the last follow-up. Major complications were defined as complications of grade 3 or higher according to the Clavien-Dindo Classification.^[9]^

### 2.3. Data extraction

Two reviewers (ZD and FL) independently performed the initial literature search and selected relevant studies based on the inclusion and exclusion criteria. Data were extracted independently by the 2 investigators. The data collection template was formulated in advance and the following was extracted: the first author, publication year, study design, country of origin, baseline characteristics of the patients (e.g., age, sample size of each group, Child-Pugh classification), mean tumor size, number of nodules, mean follow-up duration, and details about the outcome measures. Any discrepancies were resolved by discussion and consensus during the process of research selection and data extraction or by consulting the third investigator (LR) when necessary.

### 2.4. Quality assessment

The methodological quality of all the included studies was assessed by 2 reviewers (ZD and XS), with discrepancies resolved by consensus. The methodological quality of the RCTs was assessed by the Cochrane Collaboration tool for assessing the risk of bias (ROB). The total ROB of a study was considered “low” when more than 4 items associated with “low risk” by the Cochrane Collaboration ROB tool were considered applicable, “moderate” when 2 to 3 items were applicable, and “high” when fewer than 2 “low risk” items or more than 1 “high risk” item were considered applicable.^[10]^ The Newcastle-Ottawa quality assessment Scale (NOS) was used to evaluate the quality of nonrandomized trials. The overall quality of a study was defined as “poor” if the total NOS score was less than 4, “fair” if the score was 4 to 6, and “good” with a score of 7 to 9.^[11]^

### 2.5. Statistical analysis

All statistical aspects of the meta-analysis were conducted in Review Manager 5.3 (Cochrane Collaboration, Oxford, UK). Heterogeneity between included studies was assessed by means of Cochrane’s Chi-Squared test, with the significance threshold settled at 0.10, and *I*^2^ statistic, with a value of >50% being suggestive of significant heterogeneity.^[12]^ The random effect model was used when there was significant heterogeneity between studies; otherwise, the fixed effect model was used. For dichotomous outcomes, the odds ratio (OR) and the corresponding 95% confidence intervals (CI) were calculated. Additionally, funnel plots were used to visually assess the publication bias of the enrolled studies. We applied unadjusted *P* values for the significance assessment in this study, which were set at the two-tailed .05 level for hypothesis testing.

## 3. Result

### 3.1. Study selection

**Figure 1:**
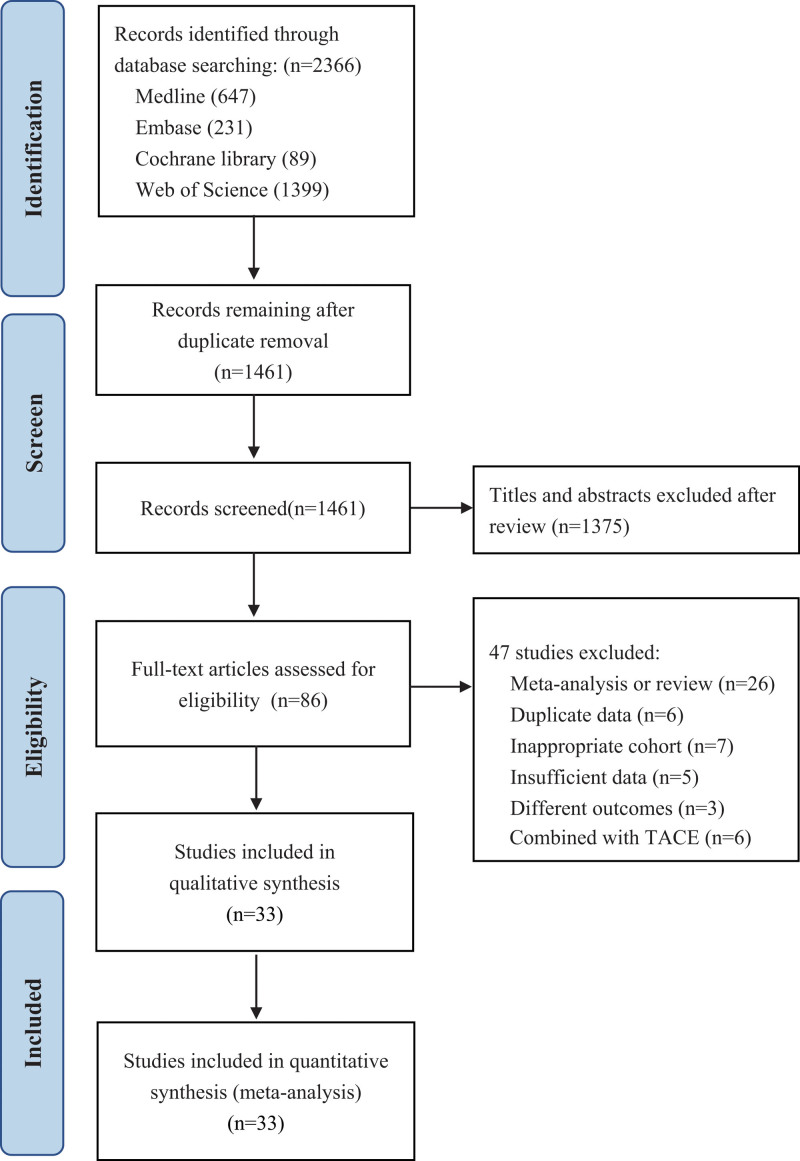
The flowchart of the study selection process for the meta-analysis.

We present the entire search process and the reasons for excluding the ineligible studies in a flowchart. Our search strategy identified a total of 3015 studies, of which 1554 studies were excluded due to duplicate data. After reviewing the abstracts and titles, 1375 studies were excluded. Fifty two studies were excluded after a full-text screening. The remaining 33 studies with a total of 4589 patients (MWA = 2 044, RFA = 2 545) were included in our final analysis^[13–45]^ (Fig. 1).

### 3.2. Characteristics of the studies and quality assessment

The 33 included studies comprised 7 RCTs,^[13,17,20,33,36,38,44]^ 24 retrospective observational trials, and 2 prospective observational trial.^[15,28]^ The sample sizes of each individual study range from 19 to 562 patients, whose age range from 50 to 69 years across studies, and male proportion rang from 38% to 94%. The recruitment period ranged from 2002 to 2019 and study follow-up range from 5 to 62 months. An overview of the characteristics of the included trials is presented in Table 1 and a summary of the risk of bias assessments of RCTs is presented in Figure S1 (Supplemental Digital Content, http://links.lww.com/MD2/B106) and Figure S2 (Supplemental Digital Content, http://links.lww.com/MD2/B107) and the quality scores of included cohort studies is presented in Table S1 (Supplemental Digital Content, http://links.lww.com/MD/G952). All the RCTs are considered low risk and all the cohort studies have a NOS score of good.

**Table 1:** Characteristics of the 33 trials included in the meta-analysis.

					Groups		NP				No. of nodules		Size (mm)		CPC (A/B/C)		Follow-up	
NO.	First author	Year	Country	Type	MWA	RFA	MWA	RFA	Ages(Y)	Male (%)	MWA	RFA	MWA	RFA	MWA	RFA	MWA	RFA
1	Abdelaziz	2014	Egypt	RCT	MWA	RFA	66	45	55	71	76	52	29 (9.7)	29.5 (10.3)	25/41/0	24/21/0	40	40
2	Chinnaratha	2015	Australia	Retrospective	MWA	RFA	25	101	62	78	31	114	NR	NR	NR	NR	14	14
3	Cillo U	2014	Italy	Prospective	MWA	RFA	28	28	64	80	NR	NR	25 (15–53)	27 (12–60)	NR	NR	24	24
4	Correa G	2014	USA	Retrospective	MWA	RFA	67	67	55	NR	127	127	NR	NR	NR	NR	18	31
5	Di Vece	2014	Italy	RCT	MWA	RFA	20	20	61	73	20	20	36 (22–69)	32 (23–64)	NR	NR	NR	NR
6	Ding J	2013	China	Retrospective	MWA	RFA	113	85	59	77	131	98	25.5 (8.9)	23.8 (8.1)	75/38/0	49/36/0	18	28
7	Hompes	2010	Belgium	Retrospective	MWA	RFA	6	13	60	47	16	13	NR	NR	NR	NR	6	NR
8	Kamal A	2019	Egypt	RCT	MWA	RFA	28	28	55	77	34	34	32.5 (9.2)	32.8 (9.1)	22/6/0	22/6/0	12	12
9	Kuang	2011	China	Retrospective	MWA	RFA	19	31	55	94	19	31	NR	NR	NR	NR	45	45
10	Lee KF	2017	China	Retrospective	MWA	RFA	26	47	60	81	28	52	37.5 (20–60)	31 (20–60)	23/3/0	42/5/0	48	53
11	Liu Y	2013	China	Retrospective	MWA	RFA	35	54	53	61	62	70	23 (10)	25 (10)	NR	NR	32	32
12	Liu W	2018	China	Retrospective	MWA	RFA	126	436	56	90	162	482	22.5 (17–29)	23 (18–30)	NR	NR	37	34
13	Lu M	2005	China	Retrospective	MWA	RFA	49	53	52	85	98	72	25 (12)	26 (12)	22/27/0	47/6/0	25	25
14	Ohmoto K	2009	Japan	Retrospective	MWA	RFA	49	34	65	80	56	37	17 (8–20)	16 (7–20)	31/14/4	20/11/3	34	26
15	Potrezzke	2016	USA	Retrospective	MWA	RFA	99	55	61	79	136	69	22 (20–23)	24 (22–26)	NR	NR	24	31
16	Qian	2012	China	Prospective	MWA	RFA	22	20	54	93	22	20	21 (4)	20 (5)	NR	NR	5	5
17	Sakaguchi	2009	Japan	Retrospective	MWA	RFA	142	249	65	71	142	249	22.8 (7.4)	24.8 (8.9)	86/56/0	147/98/4	NR	NR
18	Santambrogio R	2017	Italy	Retrospective	MWA	RFA	60	94	69	73	NR	NR	21.5 (5.3)	19.2 (5)	60/0/0	94/0/0	27	27
19	Sever IH	2018	Turkey	Retrospective	MWA	RFA	20	20	64	70	30	25	28 (10)	24 (11)	14/4/2	11/4/5	6	6
20	Shady	2017	USA	Retrospective	MWA	RFA	48	62	NR	66	60	85	17 (7–37)	18 (6–45)	NR	NR	29	56
21	Shibata T	2002	Japan	RCT	MWA	RFA	36	36	63	69	46	48	22 (9–34)	23 (10–37)	19/17/0	21/15/0	18	18
22	Simo KA	2011	USA	Retrospective	MWA	RFA	13	22	59	74	15	27	23.1 (14–39)	25.3 (12–44)	7/6/0	12/7/3	7	19
23	Sparchez Z	2019	Romania	Retrospective	MWA	RFA	17	44	61	52	20	62	25.5 (15–33)	25 (16.5–30)	NR	NR	NR	NR
24	Tian W	2014	China	RCT	MWA	RFA	60	60	55	78	79	86	26 (13)	22 (9)	NR	NR	NR	NR
25	van Tilborg	2016	Netherlands	Retrospective	MWA	RFA	15	96	61	65	32	139	25 (4–65)	24 (2–68)	NR	NR	49	NR
26	Vietti V	2018	Switzerland	RCT	MWA	RFA	71	73	66	84	98	104	18 (6.5)	18 (7.1)	57/14/0	53/20/0	26	25
27	Vogl TJ	2015	Germany	Retrospective	MWA	RFA	28	25	59	79	36	32	36 (9–50)	32 (8–45)	NR	NR	NR	NR
28	Xu HX	2004	China	Retrospective	MWA	RFA	54	43	53	86	112	78	25 (11)	26 (14)	NR	NR	NR	NR
29	Xu Y	2017	China	Retrospective	MWA	RFA	301	159	54	80	NR	NR	17 (3)	17 (3)	278/23/0	140/19/0	53	62
30	Yang B	2017	China	Retrospective	MWA	RFA	71	108	50	65	121	188	NR	NR	NR	NR	39	39
31	Yin X	2009	China	Retrospective	MWA	RFA	50	59	53	87	NR	NR	NR	NR	NR	NR	22	22
32	Yu J	2017	China	RCT	MWA	RFA	203	200	NR	NR	265	251	27 (10)	26 (10)	NR	NR	35	35
33	Zhang L	2013	China	Retrospective	MWA	RFA	77	78	54	85	105	97	22 (4)	23 (4)	77/0/0	78/0/0	25	26

### 3.3. Local tumor progression

**Figure 2.**
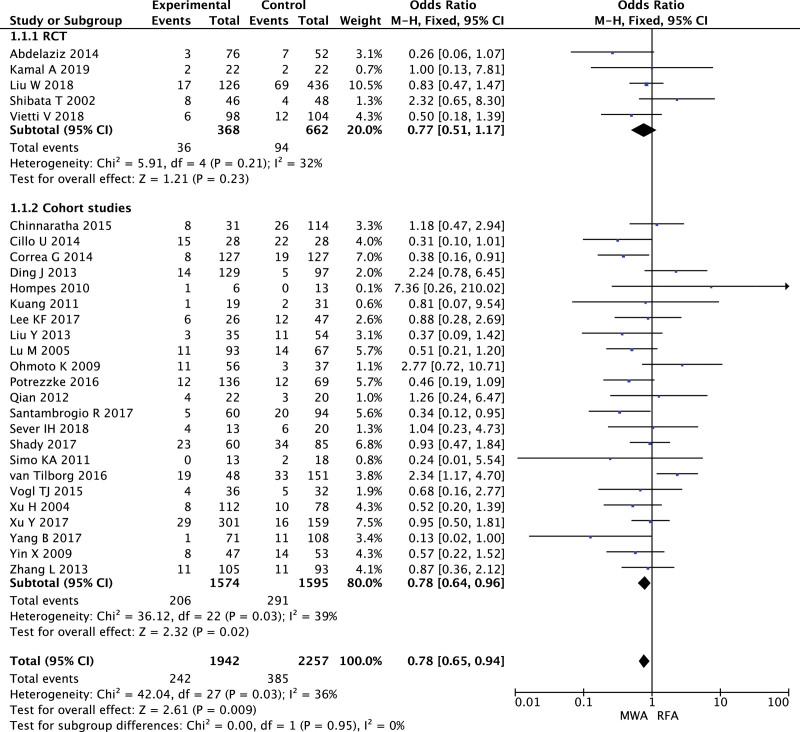
Forest plot of meta-analysis comparing local tumor progression between MWA and RFA.

LTP were reported in 28 studies, in which 5 RCTs with 1030 patients and 23 cohorts with 3169 patients. MWA had a lower LTP than RFA in cohort studies (OR = 0.78, 95% CI 0.64–0.96, *P* = .02). There was no significant heterogeneity between RCTs (*I*^2^ = 32%), as did observational studies (*I*^2^ = 39%, Fig. 2), and visual inspection of a funnel plot suggested no evidence of publication bias Figure S3 (Supplemental Digital Content, http://links.lww.com/MD2/B108).

### 3.4. Complete ablation rate

**Figure 3.**
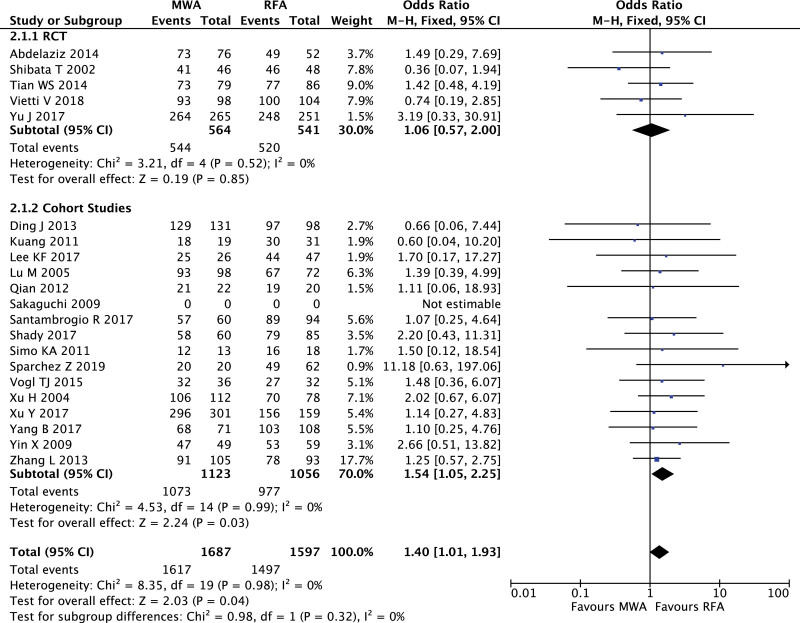
Forest plot of meta-analysis comparing complete ablation rate between MWA and RFA.

Five RCTs with 1105 patients and 16 cohort studies with 2179 patients reported CA rate, and heterogeneity and publication bias were not found in both of 2 groups (RCT: *I*^2^ = 0%; cohort studies *I*^2^ = 0%, Figure S4, Supplemental Digital Content, http://links.lww.com/MD2/B109). There was no significant difference of CA rate between MWA and RFA in RCTs (OR = 1.06, 95% CI 0.57–2.00, *P* = .85), but CA rate of MWA was higher than that of RFA in cohort studies (OR = 1.54, 95% CI 1.05–2.25, *P* = .03. Fig. 3).

### 3.5. Overall survival

We compared the overall survival of 1-year, 3-year, and 5-year in RCTs and cohort studies respectively. In RCTs, there was no significant difference in overall survival rate between MWA and RFA (1-year: OR = 1.86, 95% CI 0.91–3.80, *P* = .09; 3-year: OR = 1.16, 95% CI 0.77–1.74, *P* = .49; 5-year: OR = 0.79, 95% CI 0.51–1.21, *P* = .27), and significant difference was also not found in cohort studies (1-year: OR = 0.97, 95% CI 0.69–1.36, *P* = .85; 3-year: OR = 0.92, 95% CI 0.75–1.13, *P* = .64; 5-year: OR = 1.12, 95% CI 0.93–1.36, *P* = .22). Low grade of intergroup heterogeneity was found in 1-year overall mortality of RCTs (*I*^2^ = 52%) and 3-year overall mortality of cohort studies (*I*^2^ = 64%) respectively (Supplemental Digital Content, Figure S5, http://links.lww.com/MD2/B110, Figure S6, http://links.lww.com/MD2/B111, Figure S7, http://links.lww.com/MD2/B112).

### 3.6. Disease-free survival

We also summarized the differences of disease-free survival of in RCTs and cohort studies separately. In RCTs, there was no significant difference in disease-free survival of 1-year (OR = 1.04, 95% CI 0.48–2.24, *P* = .92) and 3-year (OR = 3.00, 95% CI 0.91–9.87, *P* = .07) between MWA and RFA. Only one study in RCTs reported the disease-free survival of 5-year, which indicates that MWA was better than RFA (OR = 1.86, 95% CI 1.20–2.86, *P* = .005). For the cohort studies, the disease-free survival of 1-year (OR = 1.20, 95% CI 0.96–1.51, *P* = .11), 3-year (OR = 1.15, 95% CI 0.93–1.41, *P* = .20) and 5-year (OR = 0.84, 95% CI 0.67–1.05, *P* = 0.13) were not found any differences. No significant heterogeneity in all the studies (Supplemental Digital Content, Figure S8, http://links.lww.com/MD2/B113, Figure S9, http://links.lww.com/MD2/B114, Figure S10, http://links.lww.com/MD2/B115).

### 3.7. Major complication

**Figure 4.**
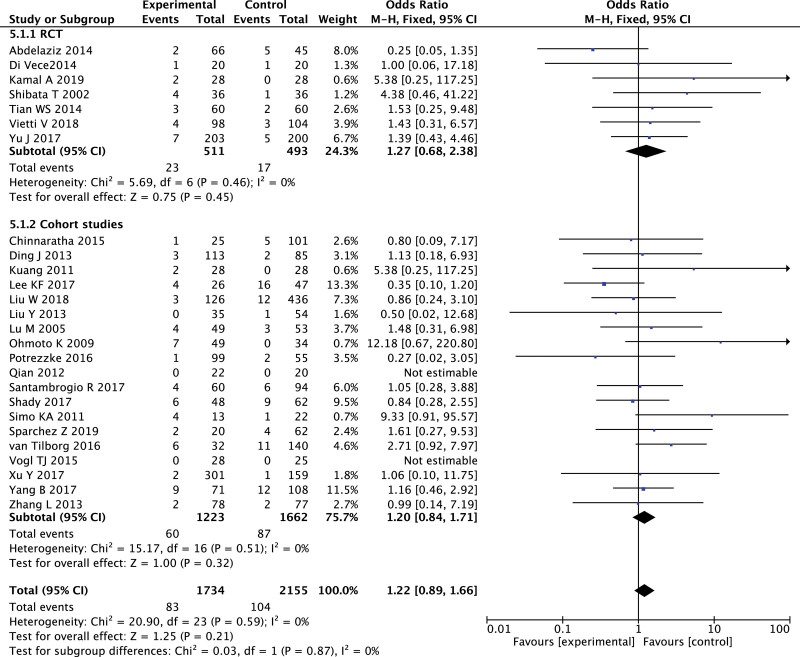
Forest plot of meta-analysis comparing major complications between MWA and RFA.

A total of 26 studies, including 3889 patients, reported major complications, with no significant heterogeneity and publication bias in each of 7 RCTs, and 19 cohort studies (Figure S11, Supplemental Digital Content, http://links.lww.com/MD2/B116). Meta-analysis showed that there was no significant difference in the main complications between MWA and RFA, whether in RCTs or in cohort studies (Fig. 4).

## 4. Discussion

This study included 33 articles with a total of 4589 patients, which includes the largest number of patients and the latest literature on the research of Microwave ablation (MWA) and Radiofrequency ablation (RFA) in hepatocellular carcinoma (HCC). Our study found that the LTP after MWA treatment was lower than that of RFA, and the complete ablation (CA) rate of MWA was higher than that of RFA. There were no significant differences in the overall survival, disease-free survival, and major complications between the 2 kinds of ablation.

Ablation therapy is considered as the first choice of treatments for most of patients with small hepatocellular carcinoma nodules, or as an alternative treatment for patients who are not suitable for surgical resection or whose chemotherapy have failed.^[46,47]^ The most commonly used ablation modalities in clinical practice are MWA and RFA.^[48,49]^ RFA is performed by advancing an especially designed electrode into the lesion and radiofrequency energy emitted from the tip of the electrode is converted into heat to create a zone of thermal destruction that encompasses the tumor, but the result is also affected by the heat-sink effect.^[50–52]^ RFA is considered the best therapeutic modality for very early and early-stage HCC according to BCLC staging when resection or liver transplantation is not indicated.^[53–55]^ MWA uses electromagnetic energy to create an electromagnetic field that heats rapidly the target tissue and induces coagulation necrosis. In comparison with RFA, MWA is more homogenous and the heat-sink effect is reduced due to the higher temperatures and the faster heating that is produced by electromagnetic energy. On the other hand, the higher elevation of temperature in the MWA field can injure the adjacent structures.^[56–58]^ Lloyd et al demonstrated rapid ablation and low morbidity in patients who underwent MWA.^[59]^ Another recent study which enrolled 221 patients showed high technique effectiveness rate and well tolerance from patients.^[60]^ Clinically, if the tumor nodule was less than 3 cm, both of the 2 methods may be considered. However, when the HCC diameter of nodules is larger than 3 cm, MWA can remove the nodules more effectively due to its higher temperature and faster heating.^[61,62]^

The treatment of patients with HCC is multidisciplinary, so nodules clearance alone cannot determine the prognosis of the patients.^[2]^ It is the reason that why no difference in overall survival between the 2 groups was found in our study and previous studies only with the treatment of ablation. Local tumor progression responds to the clinical effects of MWA or RFA earlier and more accurately. However, no difference in 1-year disease-free survival may be related to the short postoperative period and delayed local invasion development.

The major complication rate of MWA and RFA remains controversial. Major complications of RFA include intraperitoneal bleeding, infections, liver failure, pneumothorax, organ injury, bile duct stenosis and tumor lysis syndrome, but the major complication rate and procedural mortality rate is significantly low.^[63,64]^ The major complications of MWA are bleeding, peritoneal hemorrhage, liver abscess, hemothorax, colon perforation and bile duct stenosis.^[65]^ Our study including 3889 patients found no difference in the main complications between MWA and RFA, whether in RCTs or in cohort studies, so as the previous studies.^[5,6,66]^ Considering that MWA is less affected by the heat-sink effect, MWA can produce more larger tumor necrosis. However, these characteristics are in turn related to the increased risk of damaging neighboring organs, especially the structure of blood vessels. A larger ablation area might account for a higher complication rate.^[67,68]^ A defect of MWA is high local development of tumor which may be caused by a larger applicator (5 mm in diameter) applied for tumor puncture increasing the risk of bleeding and subsequent tumor seeding.^[69]^ It should be noted that RFA is performed by the guidance of ultrasound, computed tomography or magnetic resonance imaging, while MWA is performed under computed tomography or ultrasound guidance, so the types of devices and the experience of the operators would also affect the results, and high-quality evidence is needed to compare the complication rates of MWA and RFA.

## 5. Limitations

First, 24 of the 33 included studies were retrospective studies, in which the lack of randomization of patient grouping may affect the results of the study. However, the basic characteristics of each study included were not statistically different, so our results are still credible. Second, different types of generators and antennas were used in RFA and MWA in the included studies, while different stages, equipment, and experiences of operators may affect the treatment effects. Third, there are differences in the observation time points in the definition of CA and LTP. For example, complete tumor ablation should be assessed by imaging ideally 1 week to 1 month after the procedure and no later than 3 months afterwards in guidelines.^[70]^ The evaluation of treatment effect at different observation times will inevitably lead to differences. Finally, most of the studies conducted in a single center, and the number of patients involved is small, which results in heterogeneity among some studies.

## 6. Conclusion

Our meta-analysis showed that Microwave ablation has higher complete ablation and lower local tumor progression than Radiofrequency ablation in the ablation treatment of hepatocellular carcinoma nodules. There was no significant difference in overall survival between the 2 treatments. Taking into account the differences in equipment and operator experience in the included studies, high-quality randomized controlled trials are needed to draw a conclusion on the pros and cons of Microwave ablation and Radiofrequency ablation.

## Author contributions

Conceptualization: Zhimin Dou, Bin Li, Xun Li.

Data curation: Fei Lu, Zhimin Dou

Formal analysis: Fei Lu.

Funding acquisition: Zhimin Dou.

Investigation: Zhimin Dou, Fei Lu, Xiaojing Song, Xun Li.

Methodology: Longfei Ren, Xiaojing Song, Zhimin Dou.

Project administration: Zhimin Dou, Xun Li.

Software: Zhimin Dou, Fei Lu.

Supervision: Xun Li

Validation: Xiaojing Song

Visualization: Bin Li

Writing – original draft: Zhimin Dou, Bin Li, Fei Lu.

Writing – review & editing: Longfei Ren, Xiaojing Song, Xun Li, Zhimin Dou.

## Supplementary Material


