# Supplementary material for: Efficacy and safety of microwave ablation and radiofrequency ablation in the treatment of hepatocellular carcinoma: A systematic review and meta-analysis
Source: Medicine (Baltimore). 2022 Jul 29;101(30):e29321. doi: 10.1097/MD.0000000000029321 (PMC9333547; doi:10.1097/MD.0000000000029321)
Supplement: Supplementary file 12 [file medi-101-e29321-s012.docx]

Table S1. Quality scores of included cohort studies.

| Reference | Selection | | | | Comparability | Outcome | | | Score |
| --- | --- | --- | --- | --- | --- | --- | --- | --- | --- |
|  | 1 | 2 | 3 | 4 | 5 | 6 | 7 | 8 |  |
| Chinnaratha 2015 | 1 | 1 | 1 | 1 | 2 | 1 | 1 | 0 | 8 |
| Cillo U 2014 | 1 | 1 | 1 | 1 | 2 | 1 | 1 | 1 | 9 |
| Correa G 2014 | 1 | 1 | 1 | 1 | 2 | 1 | 1 | 1 | 9 |
| Ding J 2013 | 1 | 1 | 1 | 1 | 2 | 1 | 1 | 1 | 9 |
| Hompes 2010 | 1 | 1 | 1 | 1 | 2 | 1 | 1 | 0 | 8 |
| Kuang 2011 | 1 | 1 | 1 | 1 | 2 | 1 | 1 | 0 | 8 |
| Lee KF 2017 | 1 | 1 | 1 | 1 | 2 | 1 | 1 | 1 | 9 |
| Liu Y 2013 | 1 | 1 | 1 | 1 | 2 | 1 | 1 | 1 | 9 |
| Liu W 2018 | 1 | 1 | 1 | 1 | 2 | 1 | 1 | 1 | 9 |
| Lu M 2005 | 1 | 1 | 1 | 1 | 2 | 1 | 1 | 1 | 9 |
| Ohmoto K 2009 | 1 | 1 | 1 | 1 | 2 | 1 | 1 | 1 | 9 |
| Potrezzke 2016 | 1 | 1 | 1 | 1 | 2 | 1 | 1 | 1 | 9 |
| Qian 2012 | 1 | 1 | 1 | 1 | 2 | 1 | 0 | 1 | 8 |
| Sakaguchi 2009 | 1 | 1 | 1 | 1 | 2 | 1 | 1 | 1 | 9 |
| Santambrogio R 2017 | 1 | 1 | 1 | 1 | 2 | 1 | 1 | 1 | 9 |
| Sever IH 2018 | 1 | 1 | 1 | 1 | 2 | 1 | 1 | 1 | 9 |
| Shady 2017 | 1 | 1 | 1 | 1 | 2 | 1 | 1 | 0 | 8 |
| Simo KA 2011 | 1 | 1 | 1 | 1 | 2 | 1 | 1 | 0 | 8 |
| Sparchez Z 2019 | 1 | 1 | 1 | 1 | 2 | 1 | 1 | 1 | 9 |
| van Tilborg 2016 | 1 | 1 | 1 | 1 | 2 | 1 | 1 | 1 | 9 |
| Vogl TJ 2015 | 1 | 1 | 1 | 1 | 2 | 1 | 1 | 1 | 9 |
| Xu H 2004 | 1 | 1 | 1 | 1 | 2 | 1 | 1 | 0 | 8 |
| Xu Y 2017 | 1 | 1 | 1 | 1 | 2 | 1 | 1 | 1 | 9 |
| Yang B 217 | 1 | 1 | 1 | 1 | 2 | 1 | 1 | 0 | 8 |
| Yin X 2009 | 1 | 1 | 1 | 1 | 2 | 1 | 1 | 0 | 8 |
| Zhang L 2013 | 1 | 1 | 1 | 1 | 2 | 1 | 1 | 1 | 9 |
